# Explicit representation of protein activity states significantly improves causal discovery of protein phosphorylation networks

**DOI:** 10.1186/s12859-020-03676-2

**Published:** 2020-09-17

**Authors:** Jinling Liu, Xiaojun Ma, Gregory F. Cooper, Xinghua Lu

**Affiliations:** 1 Department of Biomedical Informatics, University of Pittsburgh, 5607 Baum Blvd, Suite 500, Pittsburgh, PA 15206 USA; 2 Present address: Department of Engineering Management and Systems Engineering and Department of Biological Sciences, Missouri University of Science and Technology, Rolla, MO 65409 USA

**Keywords:** Causal inference, Protein kinase activity state, Protein phosphorylation networks, Cancer signaling pathways

## Abstract

**Background:**

Protein phosphorylation networks play an important role in cell signaling. In these networks, phosphorylation of a protein kinase usually leads to its activation, which in turn will phosphorylate its downstream target proteins. A phosphorylation network is essentially a causal network, which can be learned by causal inference algorithms. Prior efforts have applied such algorithms to data measuring protein phosphorylation levels, assuming that the phosphorylation levels represent protein activity states. However, the phosphorylation status of a kinase does not always reflect its activity state, because interventions such as inhibitors or mutations can directly affect its activity state without changing its phosphorylation status. Thus, when cellular systems are subjected to extensive perturbations, the statistical relationships between phosphorylation states of proteins may be disrupted, making it difficult to reconstruct the true protein phosphorylation network. Here, we describe a novel framework to address this challenge.

**Results:**

We have developed a causal discovery framework that explicitly represents the activity state of each protein kinase as an unmeasured variable and developed a novel algorithm called “InferA” to infer the protein activity states, which allows us to incorporate the protein phosphorylation level, pharmacological interventions and prior knowledge. We applied our framework to simulated datasets and to a real-world dataset. The simulation experiments demonstrated that explicit representation of activity states of protein kinases allows one to effectively represent the impact of interventions and thus enabled our framework to accurately recover the ground-truth causal network. Results from the real-world dataset showed that the explicit representation of protein activity states allowed an effective and data-driven integration of the prior knowledge by InferA, which further leads to the recovery of a phosphorylation network that is more consistent with experiment results.

**Conclusions:**

Explicit representation of the protein activity states by our novel framework significantly enhances causal discovery of protein phosphorylation networks.

## Background

Protein phosphorylation networks play a significant role in cellular signaling under physiological and pathological conditions [1]. Many drugs are specifically designed to block signals originating from aberrantly activated protein kinases [2, 3]. A better understanding of protein phosphorylation networks will enhance our understanding of cellular signaling systems and facilitate design of new drugs.

In essence, a protein phosphorylation network is a causal network. In such networks, phosphorylation of a protein kinase usually leads to its activation, and the activated kinase will in turn phosphorylate downstream target proteins [4]. It is the kinase activity state, rather than its phosphorylation state, that drives the causal chain among different protein kinases. To discover such causal networks, researchers often systematically apply genetic or pharmacological perturbations to a cellular system to resolve the causal relationships among the proteins of interest [5, 6]. There has been a rapid accumulation of phosphorylation level data in which the levels of the phosphorylated forms of proteins are measured [7]. Researchers commonly apply causal inference approaches to such data to infer protein phosphorylation networks. However, direct use of phosphorylation level data may be insufficient or even misleading, because phosphorylation levels do not necessarily reflect the activity states of protein kinases. Particularly when a cellular system is subjected to genetic or pharmacological perturbations (e.g., activating mutations of kinases in cancers or pharmacological agents blocking activities of kinases) the phosphorylation status of a kinase and its target protein can be decoupled. Under such circumstances, conventional approaches of attempting to learn causal relationships among phosphoproteins solely based on protein phosphorylation status may fail.

To address such challenges, we have designed a novel causal discovery framework in which the phosphorylation status of each protein kinase is represented as a measured variable and its activation status is explicitly represented as a latent variable; the statistical relationships between the phosphorylation status of a protein kinase and its activity is represented as a stochastic relationship. The impact of interventions on kinase activity can thereby be explicitly represented. Moreover, prior knowledge can be integrated so that inference of kinase activity states is both knowledge-guided and data-driven. We designed a learning framework that iteratively infers the activity states of protein kinases. Through a series of simulation experiments and an application to a real-world data set, we found that explicitly representing the activity states of protein kinases can help identify a more accurate protein phosphorylation network.

## Methods

### The novel causal discovery framework

**Fig. 1:**
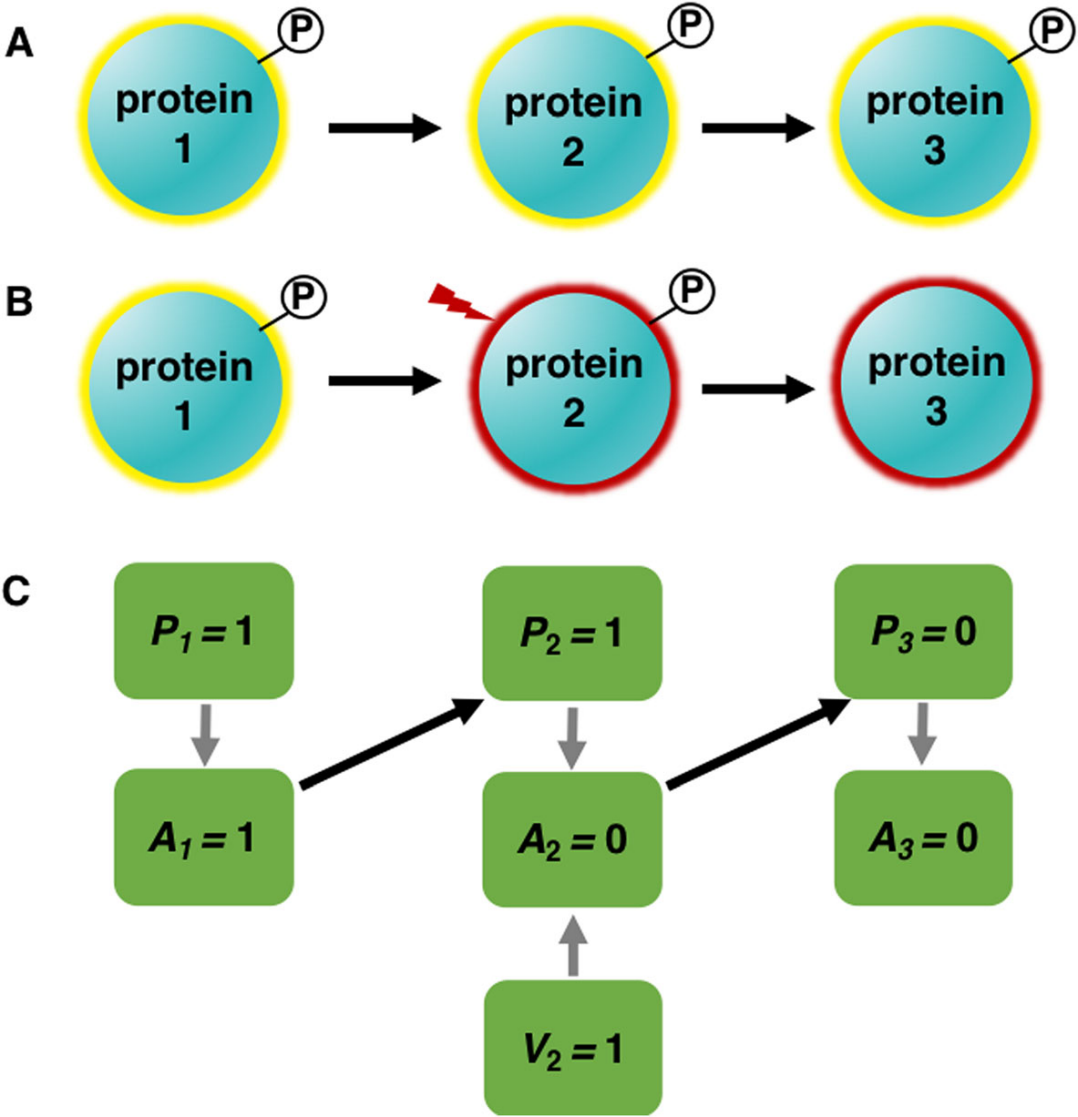
The novel causal discovery framework. **a** A phosphorylation network without interventions. A yellow glow indicates the protein is active while ‘p’ in the circle shows the protein is phosphorylated. **b** A phosphorylation network with an intervention. A red glow suggests the protein is inactive. **c** The novel causal discovery framework. *P* nodes represent protein phosphorylation states; *A* nodes represent protein activity states; *V* nodes represent the intervention states. Gray arcs are required default arcs and black arcs represent the protein causal relationships to be inferred

As an example, we use a simple phosphorylation network containing three proteins to illustrate our framework. In this system, there are three types of variables (nodes) representing quantities of interest. A “*P*” node in Fig. 1 represents the phosphorylation state of a kinase, which can be measured by its phosphorylation level. If protein2 is phosphorylated as indicated by its phosphorylation level, protein2 likely becomes active, and it will further phosphorylate protein3 to likely activate protein3 (Fig. 1a). However, when protein2’s kinase activity is inhibited by an inhibitor that does not affect protein2’s phosphorylation state, protein2 is inactive even though it is phosphorylated, and as such it cannot further phosphorylate protein3 (Fig. 1b). In this case, the phosphorylation states of protein2 and protein3 are decoupled. If we represented each protein only with its phosphorylation state (a *P* node), it would be difficult to learn the true causal relationship between protein2 and its downstream protein3, when such perturbation events occur. We propose a novel causal discovery framework where each protein is represented by both its phosphorylation state (a *P* node) and its activity state (an *A* node), an unmeasured latent variable. Figure 1c illustrates this representation for the example. A default causal arc from the *P* node to the *A* node for each protein is required, since the protein phosphorylation state can affect its activity state (Fig. 1c). If a protein is intervened upon, an intervention (*V*) node representing the intervention action will be included in the model, and a causal arc from this *V* node to the protein’s activity node will be added (Fig. 1c) since the intervention can directly affect the protein activity state. Finally, the activity state of a kinase causally influences the phosphorylation states of its downstream targets (Fig. 1c). This causal discovery framework models more accurately the underlying biological process, and it allows us to effectively represent the impact of interventions on protein activity states, as well as model the stochastic relationships between protein phosphorylation and activity states using causal Bayesian network (CBN) discovery methods.

### Overview of CBN methods

A CBN is a probabilistic graphical model that contains a graphical structure and a set of parameters. The graphical structure is represented by a directed acyclic graph (DAG) which contains nodes and directed edges. Nodes represent domain variables and directed edges represent the causal relationships between variables. A set of parameters contains the conditional probability of each node given its parents. The joint probability of all the nodes in a CBN can be written as a product of each node’s conditional probability, based on the causal Markov assumption [8]. A CBN is a flexible framework for modeling a complex domain by representing the joint probability distribution of variables, which allows unobserved variables and stochastic elements during network inference. A learned CBN with directed edges can reveal the causal relationships among variables [9]. CBNs are typically learned by two approaches: constraint-based and score-based methods. Constraint-based methods, such as the PC algorithm, use statistical tests of conditional independence to learn a CBN structure [10], which is relatively efficient, but sensitive to error propagation. Score-based algorithms use a score, such as a Bayesian posterior probability, to search for causal models with the highest score [9]. As compared to traditional constraint-based algorithms, score-based ones can assign a relative confidence score to a model, enabling selection among candidate models. In this study, we used a state-of-the-art score-based method, the FGES algorithm, which can efficiently analyze data sets containing tens of thousands of variables and performs well on simulated data sets [11].

### Simulated data

**Fig. 2:**
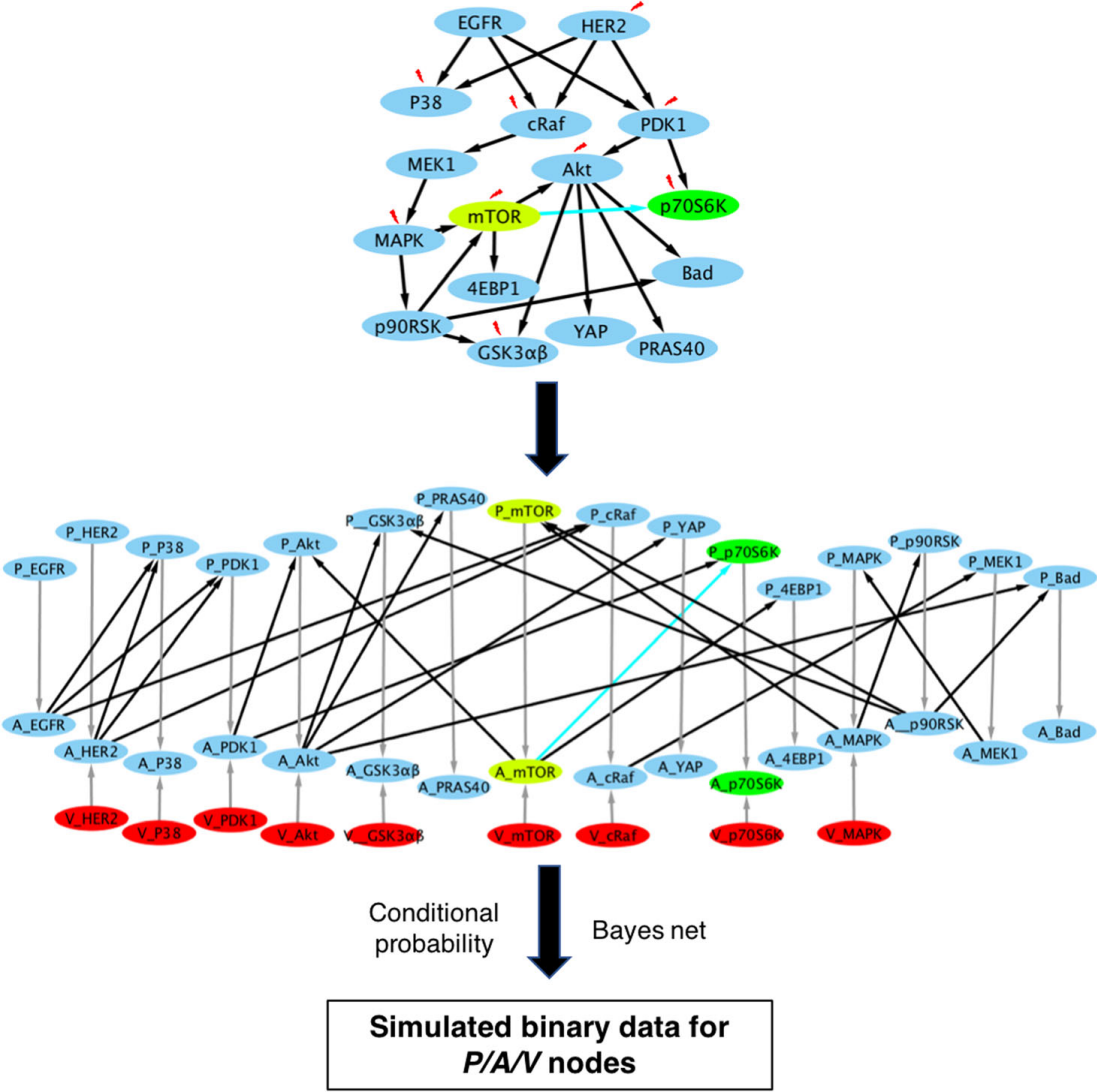
Outline of the simulation approach. The top graph is the ground-truth network where red lighting represents the applied interventions. The middle graph is the ground-truth network represented by our framework, where gray arcs are the required default arcs, black arcs come from the ground-truth network above and red nodes represent intervention nodes. Nodes for mTOR are highlighted in fluorescent yellow, nodes for p70S6K are highlighted in green, and the arc between them is highlighted in cyan. These highlights were for better visualization of the transformation from the ground-truth network to the middle network represented by our causal discovery framework

To illustrate the potential impact of systematic perturbations in learning protein phosphorylation networks, we performed a series of simulation experiments. Based on prior knowledge of a protein phosphorylation network of 35 protein kinases that came from literature and also applied to the real-data to be introduced below [6], we created a “ground-truth” causal network of 16 kinases, which consists of all three types of nodes (Fig. 2). In this network, each kinase is represented by two types of nodes: protein phosphorylation states (*P* nodes) and kinase activity states (*A* nodes). For each kinase, there is a causal arc from its *P* node to its *A* node, and some or no causal arcs between its *A* node and the *P* nodes of other proteins, depending on the prior knowledge. In this simulated experiment, we set *P* (*A* = 1 | *P* = 1) = 1, as well as P (*A* = 1 | *P* = 0) = 0 if there is no intervention. We further simulated the impact of interventions on 9 proteins by adding a set of *V* nodes reflecting the states of inventions and by directing these *V* nodes to the corresponding *A* nodes; when a *V* node is set to “1”, it directly inhibits the activity state of the corresponding *A* node (i.e., set the activity of the kinases to “inactive” or “0” state), without changing the protein’s phosphorylation state (Fig. 2). Under these conditions, an *A* node integrates both information from its *P* node and *V* node: it will be a copy of *P* node if there is no intervention, and it will be set to “0” if there is an intervention. With this CBN, we used the Tetrad causal modeling and discovery system (https://www.ccd.pitt.edu/tools/) to instantiate the parameters of conditional probabilities and to further simulate binary values for all the *P* nodes, *V* nodes, and *A* nodes for 500 instances (Fig. 2).

### Real data: protein phosphorylation data and preprocessing

The proteomic data set used in this study involved the perturbation of four different human breast cancer cell lines and measurement of the abundance of phosphoproteins [6]. The breast cancer cell lines included BT20(Basal), BT549(Claudin-low), MCF7(Luminal) and UACC812(HER2+), which were pre-treated using five different inhibitors and DMSO (as a control) for 2 hours and then stimulated by eight different stimuli. The interventions as well as their potential targets are summarized in Additional file 1. Reverse phase protein array (RPPA) assays were performed to measure the abundance of 35 phosphoproteins harvested at seven different time points within a four-hour period. The amounts of phosphorylated proteins (phosphorylation level), rather than the total protein abundance, were measured in the data set used here.

We log-transformed and discretized the continuous data for the abundance of 35 phosphoproteins that was downloaded from [6]. For each cell line, we extracted all the cases treated with DMSO and collected at time 0, a condition where cells are not perturbed by inhibitors nor stimuli. These cases served as controls. We compared all the other cases to the control group to examine whether there was any significant difference in phosphorylation levels. If the value of a protein at a non-control condition is significantly different from those derived under the control condition (*p*-value < 0.05), we set the phosphorylation state of the protein under the condition to 1; otherwise, a discretized value of 0 was used, indicating no changes of the protein phosphorylation states compared to the control condition. After discretizing all the cases for each cell line, we combined all the discretized values from all four cell lines to form a larger dataset for further analysis. If a protein was intervened upon by pharmacological agents, its intervention was represented as a binary variable and its value was set to 1.

### Inference of protein activity states

Since the activity state of each kinase of interest is not measured (latent) under the experimental conditions in which the data were collected [6], we designed a Markov chain Monte Carlo (MCMC) algorithm [12] to infer the protein activity states; we call it the InferA algorithm. Following our causal discovery framework (Fig. 1c), we instantiate the phosphorylation state (*P* node) of each protein based on the observed phosphorylation level data. If a protein is targeted by a drug in the experiments, we also created a *V* node and instantiated its value based on the experimental conditions. An initial network was constructed as the structure to connect the *A* nodes and *P* nodes for the 35 proteins of interest (described below in more details). If there was an arc from Protein1 to Protein2 in the initial network, we added a causal arc from the *A* node of Protein1 to the *P* node of Protein2.

**Fig. 3:**
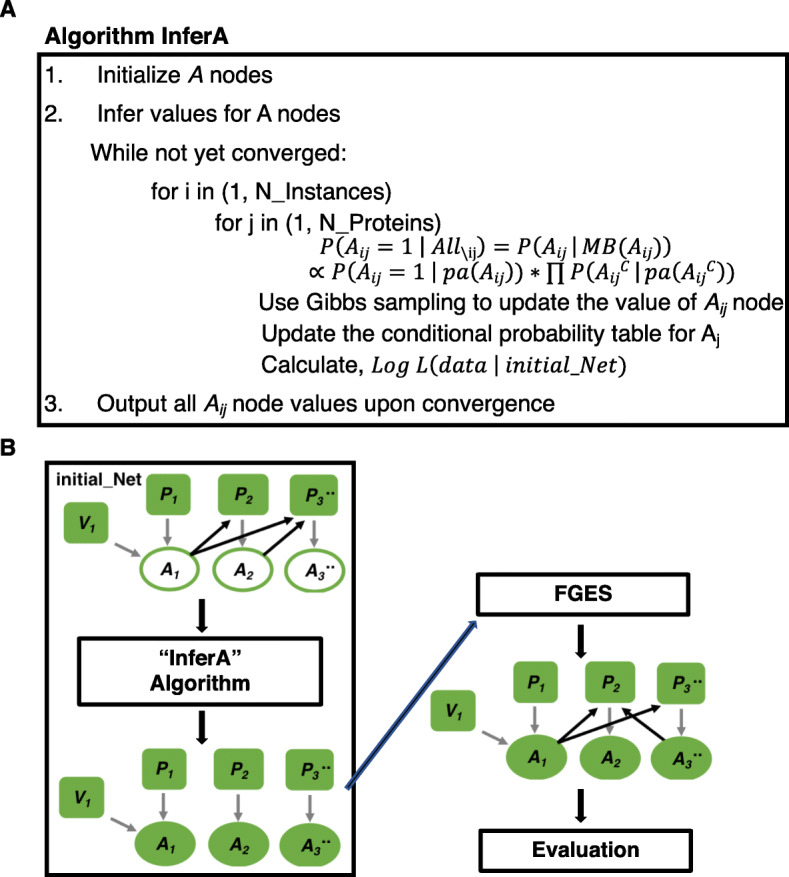
Overview of the InferA algorithm and its use with FGES. **a** The pseudo code of algorithm InferA. “*All*_*\*ij_” refers to all the nodes except *A*_*ij*_, “pa(*A*_*ij*_)” refers to the parents of *A*_*ij*_, and “*A*_*ij*_^*c*^” represents a child node of the *A*_*ij*_ node. “initial_Net” refers to the initial network, which is discussed in the subsection of “construction of initial networks” within the methods section. **b** The pipeline of InferA-FGES for predicting the causal network among phosphoproteins. Within the three networks shown in the pipeline, *P* nodes represent protein phosphorylation states; *A* nodes represent protein activity states; the gray edges are the default edges between each protein’s *P* node to its *A* node while the black edges represent the causal relationships among proteins; the black edges in the initial_Net were provided to InferA for inferring the *A* node values while the black edges in the predicted network outputted by FGES were learned by the pipeline. The unfilled ovals represent *A* nodes with unknown values while the filled ovals or squares denote nodes with known values

We inferred the conditional probability of the A node for each protein based on its Markov blanket in the initial network, as shown in the pseudo code below. The Markov blanket of a node includes the node’s parents, children, and the parents of its children, which represent all the information needed for inferring the value of this node [13]. We then used Gibbs sampling, a MCMC algorithm, to assign the value of 1 or 0 to each protein’s A node based on its conditional probability. We sampled a random number from (0,1), and compared this number with an A node’s conditional probability given its Markov blanket. If an A node’s conditional probability is smaller than this random number, then the value of this A node was set as 0; otherwise the value of this A node was set as 1. We calculated the logarithm of the joint probability for all the observed variables (*P* nodes and *V* nodes) in all the cases as a convergence criterion. We repeated sampling (E-step) and updating the conditional probability Table (M-step) until a predefined maximum number of iterations when the logarithm of joint probability converged with a small-range oscillation. Upon convergence, the resulting values for all the *A* nodes were outputted and used in the subsequent analysis. The InferA algorithm is coded in C++ and is very efficient. For example, it takes 48 s to output *A* node values for 35 *P* nodes with about 1000 instances after 1000 iterations on a server of 2 CPUs with 6 cores per CPU. The pseudo code is showed by Fig. 3a.

### Construction of initial networks

Inferring the values of the *A* nodes requires an initial structure of a CBN. We adopted two approaches to define network structures to be used in the InferA algorithm. First, a network describing the known causal relationships between the proteins of interest was curated from the literature [6], which included cycles in regulation relationships. We turned this network into an acyclic causal graph by removing a minimal number of arcs to break all cycles, and we refer to this network as the knowledge-based network (K_Net). Second, we created an initial network based on the observed data from [6] using a generalized linear regression model, and we refer to this network as data-derived network (D_Net). More specifically, we first instantiated *A* node values with the same value of the corresponding *P* node values if there was no intervention. If there was intervention for a protein, then its *A* value was set as 1. Based on the assumption that the phosphorylation state of a protein is affected by the activity states of other kinases, we performed a series of regression analyses, where a *P* node of a protein is treated as a dependent variable and the *A* nodes of other proteins as independent variables. We applied elastic network regression using the R package “glmnet.” We then constructed an initial causal network by adding causal arcs from the top *A* nodes (the absolute values of their coefficients in the regression model > 0.5) to a *P* node. Cycles in the D_Net were eliminated by removing a minimal number of arcs. Finally, using the function of Random Graph provided by the Tetrad program, we generated a DAG of random network of the same number of nodes and arcs as K_Net by randomly adding causal arcs between *A* nodes and *P* nodes. We refer this network as rand_Net, which serves as a control of a network without any biological information.

### Network learning using FGES

Causal-cmd is a command-line tool (https://www.ccd.pitt.edu/) that allows a user to call many of the causal discovery algorithms implemented in Tetrad, including FGES. FGES is an optimized and parallelized version of the greedy equivalence search (GES) algorithm [14], which can efficiently learn CBN structure on thousands of variables and samples [11].

After inferring the state of *A* nodes, we combine observed *P* node data with inferred *A* nodes as input to FGES to search for causal arcs from *A* nodes to non-self *P* nodes (Fig. 3b). FGES allows a user to set constraints on search scope. Based on our causal discovery framework (Fig. 1c), we require FGES to instantiate a default arc from the *P* node to the *A* node for each protein as well as a default arc from the *V* node to the *A* node, if there is an intervention; we prohibit FGES from adding direct edges among *P* nodes; we also prohibit FGES from adding direct edges among *A* nodes; we only allow FGES to search for edges from *A* nodes to non-self *P* nodes (Fig. 3b). To estimate the posterior probability that FGES assigns an edge between two nodes, we employed a bootstrap approach, where FGES was applied to 100 different datasets sampled from the original dataset with replacement. The reliability of each edge is estimated based on the frequency of discovering this edge over the 100 bootstrap samples.

### Evaluation of the learned protein causal networks

We have used two different approaches to evaluate the networks learned by the FGES algorithm. For the first approach, we compared the learned network to a ground-truth network. In this method, we only included edges with an estimated posterior probability > 0.5. We assessed the ability of FGES to discover the ground-truth causal network structure from three perspectives: 1) “*arc*”, which assesses how well the directed edges in the ground-truth network are recovered; 2) “*adjacency*”, which assesses how well true neighbor nodes are connected; and 3) “*path*”, which assesses how well directed paths connecting pairs of nodes are recovered. For each of the above measures, we derived the precision rate, recall rate, and their harmonic average (F1 score) for the learned network.

For the second approach, we used the evaluation criterion published by Hill et al. (2017). In their study, the mTOR inhibitor was applied to cell lines; those proteins that exhibited significant changes in phosphorylation status were identified, and they were regarded as the gold-standard descendants of mTOR. For a learned network, if there was a directed path from mTOR to a protein, we treated the protein as a descendant of mTOR. We compared the descendants of mTOR discovered by our approach to the gold-standard descendants to acquire precision and recall rates. We also calculated the area under the ROC curve (AUROC) by varying the thresholds of the learned arc probability.

## Results

We applied our causal discovery framework to both simulated and real-world data sets. We investigated whether our framework allowed a better illustration of intervention effects as well as a good usage of prior knowledge, and whether these helped obtain a more accurate protein causal network compared to the current model of using the phosphorylation levels alone.

### Simulation experiments

To investigate the impact of perturbations on disrupting statistical relationships between protein phosphorylation data and the performance of our causal discovery framework in learning protein phosphorylation networks, we performed a series of simulation experiments using CBNs with *P/A/V* nodes where 9 out of 16 protein kinases were intervened (Fig. 2). We generated samples in which the values of all nodes (*P/A/V*) were instantiated based on the joint probability defined by the structure and parameters of the CBN. Please note in the simulation experiments, the A node values were simulated based on predefined structure and parameters, not inferred by InferA. We further conducted the following experiments. First, to evaluate the impacts of perturbations on the statistical relationships among *P* nodes, we applied FGES model to infer causal network structures using just the data corresponding to *P* nodes, which is referred as the P-only experiment. Second, in previous studies [5, 15], in order to represent the impact of interventions on the systems, many researchers changed the protein phosphorylation state to a predefined value if the protein was intervened upon. While this approach helped to maintain the statistical relationships of phosphorylation states between a perturbed protein and its downstream proteins, it would disrupt the statistical relationships of its phosphorylation status with respect to its upstream proteins. To examine the impact of such approach, we produced a dataset, in which a *P*-node value was set to 0 if it was intervened upon in the sample, and we call this experiment as the **I**ntervened on ***P*** (I-P) experiment. Finally, we applied FGES to both P nodes and A nodes to search for causal arcs from *A* nodes to non-self *P* nodes, and we call this experiment as the A-P experiment.

**Fig. 4:**
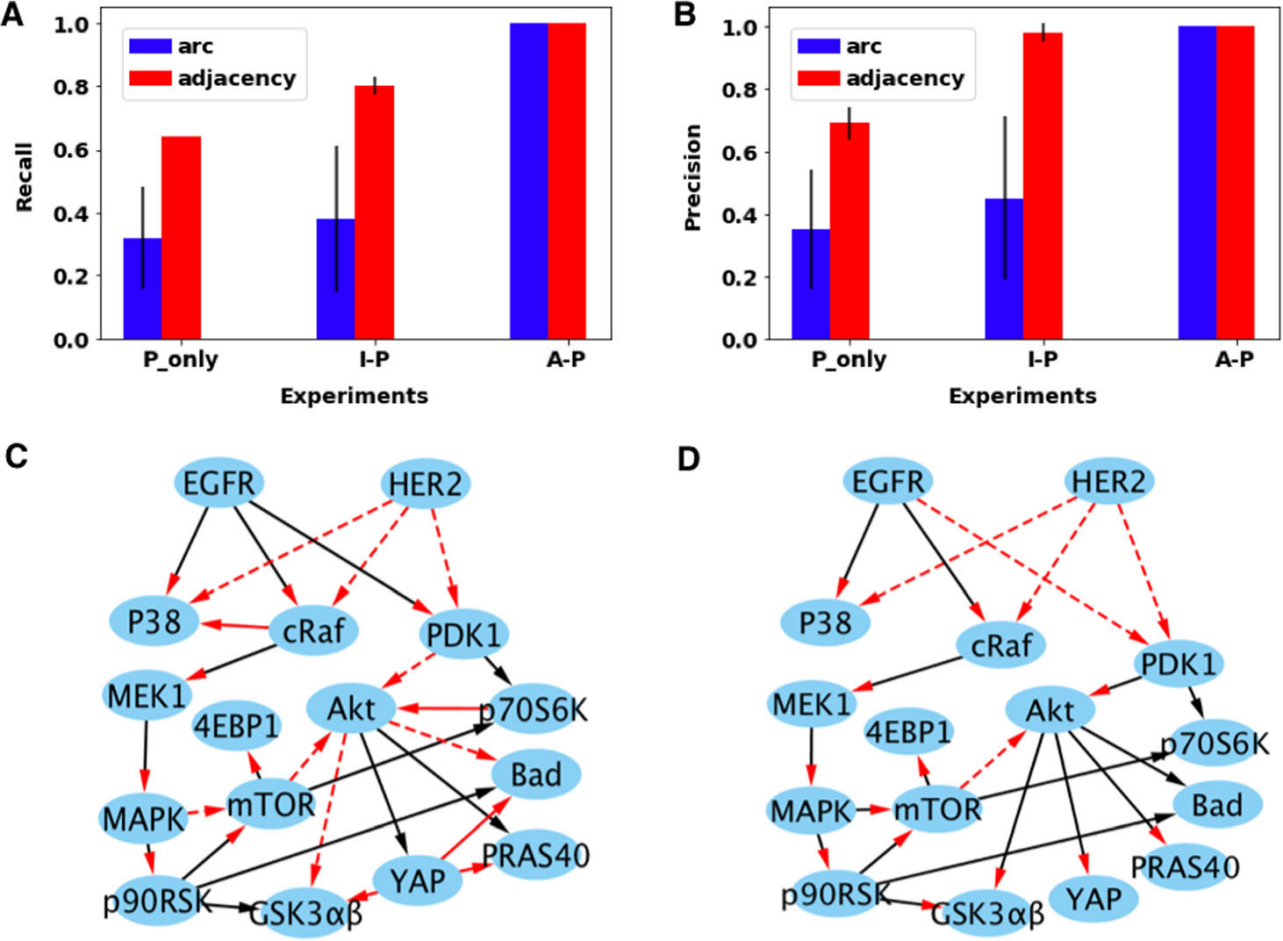
Explicit representation of protein activity states helped acquire a more accurate network with simulated datasets. **a** Recall relative to the ground-truth network. Error bars in this figure represent standard deviations from three simulation experiments. **b** Precision relative to the ground-truth network. **c** The comparison between the learned network by P_only experiment and the ground-truth network. **d** The comparison between the estimated network by I-P experiment and the ground-truth network. Dashed red arcs represent missed true arcs, solid red arcs show false arcs added, black edges with red arrowheads represent the misoriented edges, and black edges with black arrowheads show the true arcs discovered by the P_only or I-P group

In the A-P experiment from simulated data, the FGES algorithm completely recovered the ground truth network, with 100% recall and 100% precision for *arc* and *adjacency* (Fig. 4a,b). By contrast, the causal networks learned from only P nodes showed significantly lower recall (0.64) and precision (0.69) for adjacency, and much lower recall (0.32) and precision (0.35) for arcs. The I-P experiment acquired a high precision for adjacency (0.98) that is similar to the A_P experiment (Fig. 4b). But the I-P group had a worse recall for adjacency (0.8) and much lower recall (0.38) and precision (0.45) for arc, compared to the A-P experiment (Fig. 4a,b). The results provide support that under systematic perturbations, learning CBNs only based on P nodes results in relatively poor overall discovery performance. While the brute force representation of effects of interventions by discarding observed protein phosphorylation data and replacing it with a predefined value based on the interventions can partially restore the statistical relationships of phosphorylation states between an intervened protein and its downstream target proteins, the approach could not fully recover the ground truth network. Finally, inclusion of A nodes can accurately reflect the statistical relationships among the proteins and interventions, and thus achieved the complete reconstruction of the ground truth network.

We further examined the structures of the CBNs recovered by FGES from the different experiments discussed above. Compared with the ground truth network of 22 arcs, the P_only experiment included five false arcs, missed eight true arcs, failed to correctly orient eight edges and only obtained five true arcs (Fig. 4c); the I-P experiment did not add any false edges, but it removed five true arcs and failed to correctly orient thirteen edges (Fig. 4d); the A-P experiment had the same causal structure as the ground-truth network. The simulation experiments support our hypothesis that the explicit representation of protein activity states together with the protein phosphorylation states can improve the causal discovery of protein phosphorylation networks.

### InferA enables an effective integration of prior knowledge in a data-driven manner in the real-data experiments

**Fig. 5:**
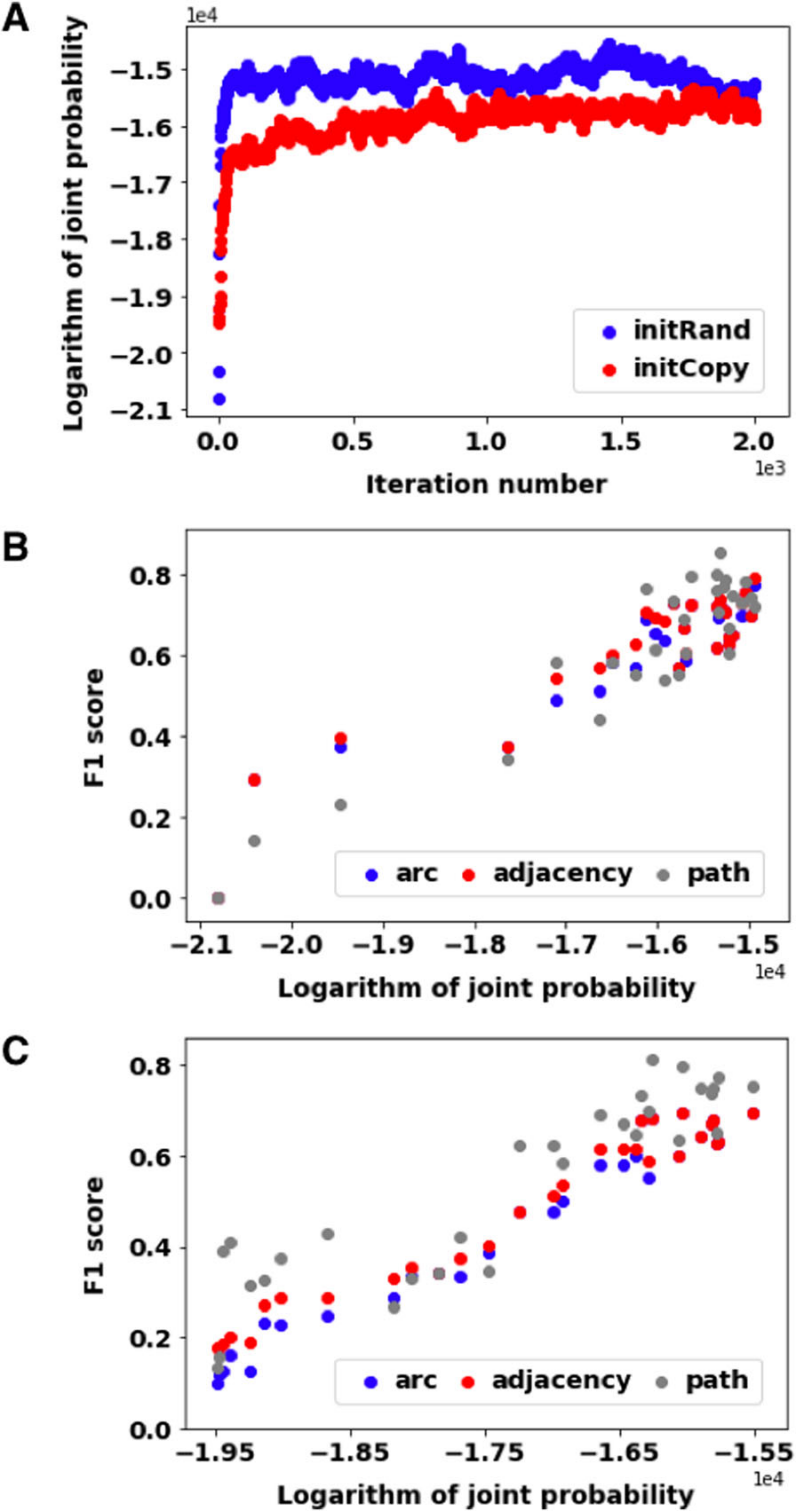
The InferA algorithm converged well for the real-world data analysis. **a** The changes of the logarithm of joint probability for observed *P* nodes and *V* nodes with iteration numbers when initializing *A* nodes with random 0/1(initRand) or initializing *A* nodes with the corresponding *P* node values (initCopy). Values on both axes are in scientific formats. **b** The F1 scores of *arc*, *adjacency* and *path* increase with the logarithm of joint probability for initRand. X-axis values are represented by scientific notations. **c** The F1 scores of *arc*, *adjacency* and *path* increase with the logarithm of joint probability for initCopy. X-axis values are shown in scientific formats

We set out to test if our framework can accurately learn causal networks based on the real-world data from [6] and evaluate whether we can recover network structures that conform with prior knowledge. Given experimental protein phosphorylation data and an initial network of K_Net that originates from a prior network curated from literature, we used our InferA algorithm to infer the *A* node values based on the observed *P/V* nodes. Within the algorithm of InferA, the *A* node values were either initialized randomly (initRand) or initialized with the corresponding *P* node values (initCopy). The logarithm of the joint probability of all the observed nodes (i.e., the *P* nodes and *V* nodes) increased as the algorithm iterated and it converged quickly for both initRand and initCopy (Fig. 5a). We outputted *A* node values at various iteration numbers before convergence. We applied FGES to the resulting *A* node values along with the observed *P/V* nodes to learn a protein-signaling causal network. Interestingly, the F1 scores for different network properties (arc, adjacency and path) exhibited positive correlations with the joint probabilities of the data under both initRand (Fig. 5b) and initCopy (Fig. 5c) situations. This positive correlation supported the convergence of the InferA algorithm to the initial network. Since initRand and initCopy worked similarly, we only show the initCopy results in the following figures.

**Fig. 6:**
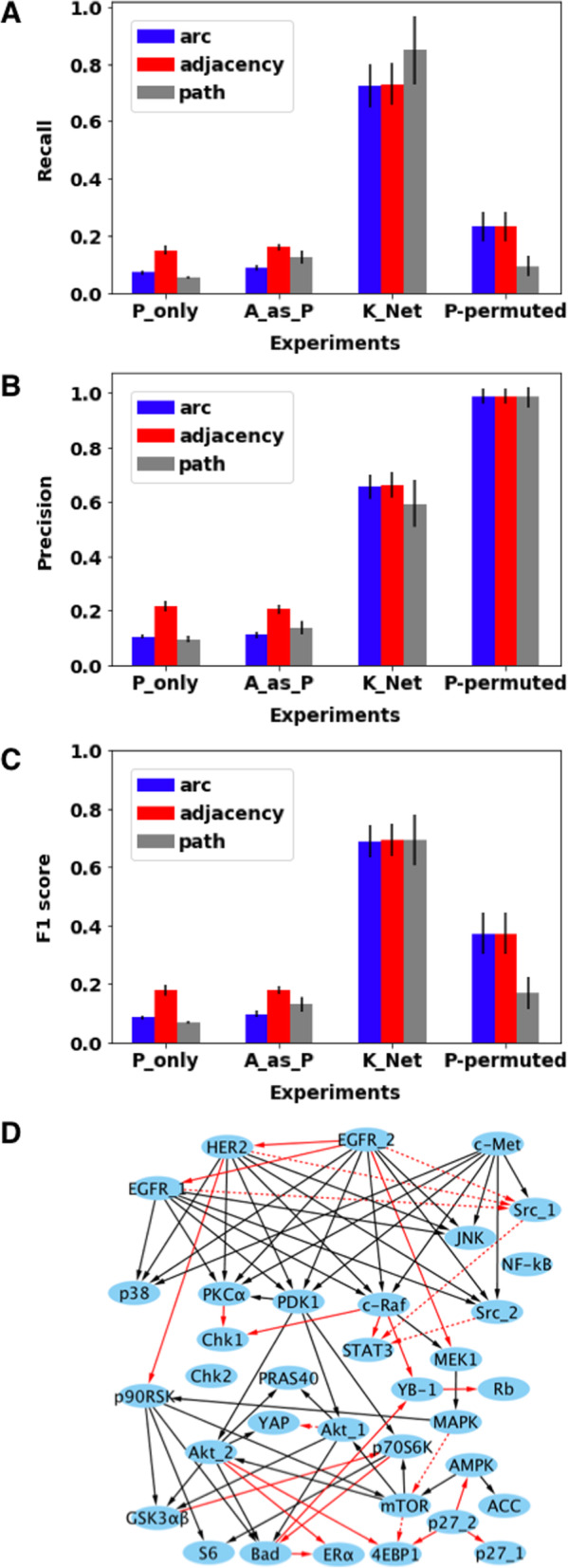
The InferA algorithm effectively integrates the prior knowledge in the real-world data analysis. Summarizes the recall **(a),** precision **(b)** and F1 score **(c)** of *arc*, *adjacency* and *path* for different experiments compared to K_Net. Error bars in this figure represent standard deviations among 50 experiments. **d** A learned protein phosphorylation network compared to K_Net. Solid black arcs showed the recovered true arcs from K_Net; dashed red arcs were the ones not recovered from K_Net; solid red arcs represent the new arcs learned by FGES

We further compared the recall, precision, and F1 score of retrieving the K_Net for the different causal networks under different experiments (Table 1). For the P_only experiment, we only used the protein phosphorylation states represented by *P* nodes to learn the protein causal network. For the other experiments, we used both *P* nodes and *A* nodes as inputs to FGES, as illustrated in Table 1. The P_only experiment had a very low recall, precision, and F1 score for *arc*, *adjacency* and *path*. The A_as_P experiment obtained similar results to the P_only experiment in discovering K_Net, indicating that simply setting A nodes to the same values as P nodes does not enhance learning. For the K_Net experiment that uses K_Net as the initial network, the recall, precision, and F1 score for *arc*, *adjacency* and *path* were all greatly improved compared to the P_only experiment (Fig. 6a, b, c). Here, the higher recall suggests that the InferA algorithm effectively integrated the prior knowledge of the protein causal interactions from K_Net. We noted that since we used K_Net as input, and compared the resulting network to K_Net, this was the best-case scenario for achieving good performance. But it was indeed an effective way of integrating the prior knowledge of K_Net. To demonstrate that the learned causal networks are data-driven (not solely determined by the prior network structure), we permuted the data values of *P* nodes and *V* nodes, and we repeated the procedures of InferA (using K_Net) and FGES network search. Indeed, the recall for *arc*, *adjacency* and *path* decreased significantly (Fig. 6a). This indicated that the effective integration of the prior network of K_Net by the InferA algorithm is data-driven. Without sensible data, it is unlikely to recover most of the causal arcs in K_Net. With disrupted statistical relationships between protein variables resulting from data permutation, FGES only reported few significant causal edges. With fewer causal edges recovered from K_Net, the precision for the P-permuted experiment became very high (Fig. 6b). The F1 scores of *arc*, *adjacency* and *path* in the P-permuted experiment were significantly lower than that in the K_Net experiment (Fig. 6c).

**Table 1:** Different experiments for the real-world data analysis

Names	Input to FGES	*A* values	Input to InferA
P_only	*P*	N/A	N/A
A_as_P	*P* + *A*	Copied *P* values	N/A
K_Net	*P* + *A*	Inferred by InferA	K_Net + *P/V*
D_Net	*P* + *A*	Inferred by InferA	D_Net + *P/V*
rand_Net	*P* + *A*	Inferred by InferA	rand_Net + *P/V*
P-permuted	permuted *P* + *A*	Inferred by InferA	K_Net + permuted *P/V*

We examined further the structure of a network learned by our framework (Fig. 6d). The learned network based on the inferred A node values from K_Net recovered many edges in K_Net. Interestingly, our methods also learned some new edges in comparison to K_Net. The biological validity of these edges remains to be examined through experiments.

### Evaluating the learned networks with respect to held-out experiment data

**Fig. 7:**
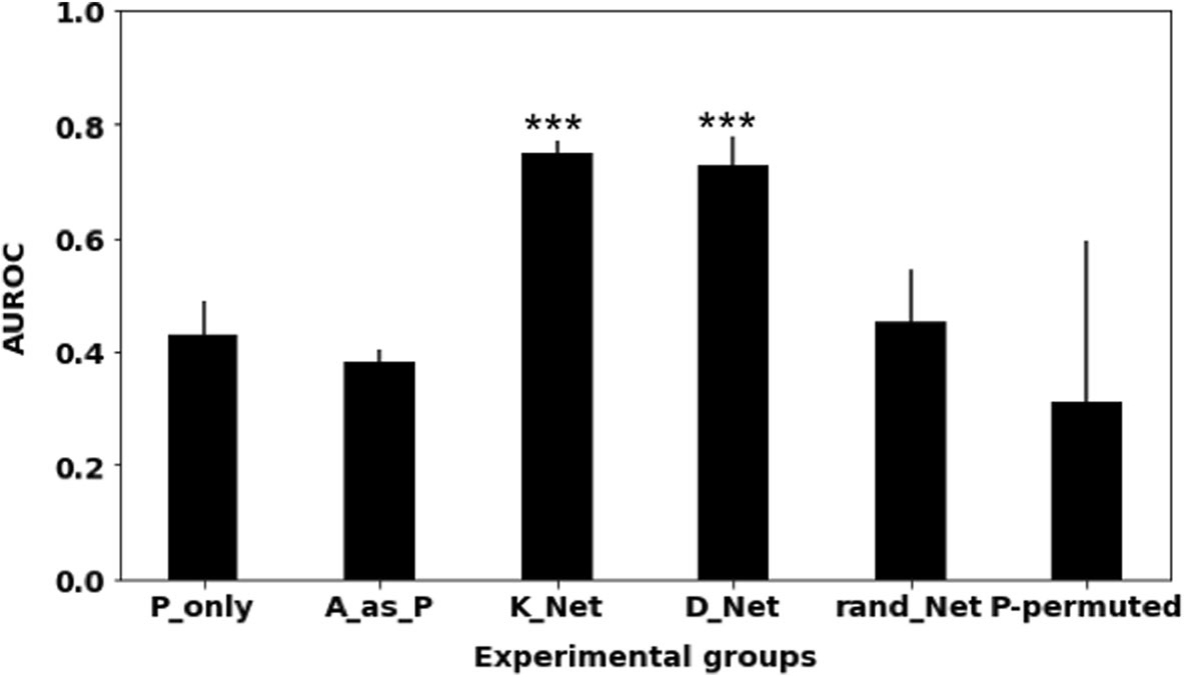
Network evaluation with respect to the held-out experiment data. AUROC results when comparing the mTOR descendants from the learned causal networks to the gold-standard ones for different experiments. Error bars in this figure represent standard deviations over 50 experiments. Three asterisks indicate significant differences (*p*-value < 0.001) compared with the P_only experiment

We then evaluated the learned causal networks against the ground truth acquired from biology experiments (i.e. gold-standard descendants of mTOR) using the same metrics and methods as described by [6]. The causal networks learned from only *P* nodes have a low AUROC of 0.43 (Fig. 7). Also, the A_as_P experiment acquired similar AUROC to the P_only experiment. The experiments using either K_Net or D_Net as an initial network for inferring the *A* node values had significantly better AUROCs (0.75 for K_Net and 0.73 for D_Net), compared to the P_only experiment. This result suggests that the explicit representation of protein activity states with *A* node values inferred from a sensible initial network helped FGES find a more accurate protein causal network. It is rather exciting to see the D_Net experiment performed very well without using any prior knowledge. With a randomly-generated initial network to infer the *A* node values, using the resulting *A* values led to a learned causal graph with a low AUROC (0.45), which supports the utility of a good initial network. If using K_Net as an initial network, but at the same time permuting *P/V* node values, FGES failed to recover true causal relationships, with an AUROC of 0.31 that is greatly decreased compared to the K_Net group (Fig. 7). This result suggested an initial prior network (i.e. K_Net) itself is not sufficient, and InferA needs both a good prior network and good data in order to derive truthful values of *A* nodes which in turn allow the discovery of a more accurate causal network of the phosphoproteins.

## Discussion

Discovering protein phosphorylation networks in specific cell types remains an important task in understanding cellular signaling systems and mechanisms of certain diseases, particularly cancer. For example, the cancer genome atlas (TCGA) project has systematically collected RPPA data of many phosphoproteins [7], and understanding how these proteins causally interact with each other under the perturbations of diverse genomic and epigenomic alterations in cancer cells could shed light on disease mechanisms of cancers. However, as illustrated in our study, attempts to infer causal phosphorylation networks solely based on the abundance of phosphoproteins are insufficient in recovering the true causal networks. We conjecture that this problem will likely be exacerbated when inferring large causal networks of many phosphoproteins under the conditions of systematic perturbations, such as in cancers where many kinases are aberrantly activated by mutations without changing their phosphorylation states. In this study, we showed that in addition to *P* nodes representing the protein phosphorylation status, including *A* nodes to explicitly represent the activity states of kinases enables one to represent the impact of interventions on protein function in a biologically sensible way, and thereby enhances the capability of CBN discovery algorithms to recover biological sensible results.

Our study illustrated important factors that may influence the discovery of causal networks. First, constructing a biologically sensible initial network, either based on prior knowledge or constructed from data, will influence the accuracy of inferring the activity states of phosphoproteins and thereby the quality of the final causal network. We showed that both K_Net and D_Net tested in our study were able to lead to sensible discoveries of the protein causal networks, whereas random initial networks did not lead to accurate causal discovery. Second, in addition to a good initial network, data are also important to achieve accurate performance, which reflects the data-driven nature of our approach. The InferA algorithm was able to effectively integrate prior knowledge of known causal relationships in a data-driven manner, which further helped FGES acquire a final causal network that is consistent with experiment results. This result reflected a strength of this Bayesian learning approach, which combines prior knowledge (in the form of an initial network structure) and data. Our learned network postulates some new causal relationships beyond the prior knowledge, which serve as hypotheses for further investigation.

## Conclusions

In summary, the results support the hypothesis that explicit representation of protein activity states together with their phosphorylation states can help causal discovery algorithms more accurately learn protein phosphorylation networks. Our InferA algorithm can estimate the protein activity states by effectively and efficiently integrating various information including the observed phosphorylation level data, interventions applied in the data, and prior knowledge of protein causal relationships.

## Supplementary information

**Additional file 1.** Interventions (stimuli or inhibitors) and their most-direct target proteins among the 35 proteins of interest in the RPPA dataset. N/A means there is either no target protein among the proteins of interest or the target proteins are not clear.

## Data Availability

Not applicable.

## Abbreviations

TCGA: The cancer genome atlas
FGES: Fast greedy equivalence search
GES: Greedy equivalence search
CBN: Causal Bayesian Network
RPPA: Reverse phase protein array
K_Net: Knowledge-based network
D_Net: Data-derived network
